# Этапность и преемственность лечения эндокринной офтальмопатии при болезни Грейвса

**DOI:** 10.14341/probl13307

**Published:** 2023-10-21

**Authors:** Е. Г. Бессмертная, А. А. Михеенков, A. С. Колодина, Т. Н. Аксенова, Д. М. Бабаева, Я. О. Груша, Н. Ю. Свириденко

**Affiliations:** Национальный медицинский исследовательский центр эндокринологии; Национальный медицинский исследовательский центр эндокринологии; Научно-исследовательский институт глазных болезней; Национальный медицинский исследовательский центр эндокринологии; Национальный медицинский исследовательский центр эндокринологии; Научно-исследовательский институт глазных болезней; Национальный медицинский исследовательский центр эндокринологии

**Keywords:** болезнь Грейвса, эндокринная офтальмопатия, оптическая нейропатия, костная декомпрессия орбиты

## Abstract

По современным представлениям, эндокринная офтальмопатия (ЭОП) — самостоятельное прогрессирующее аутоиммунное заболевание органа зрения, тесно связанное с аутоиммунной патологией щитовидной железы (ЩЖ), (код МКБ – H06.2, экзофтальм при нарушениях функции щитовидной железы Е05.0). Лечение ЭОП представляет собой длительный поэтапный процесс, включающий иммуносупрессивную терапию, лучевую терапию орбит, хирургическое лечение.

ЭОП — мультидисциплинарная проблема. Пациент с клиникой тиреотоксикоза и симптомами ЭОП будет госпитализирован в эндокринологическую клинику для нормализации тиреоидных гормонов и лечения осложнений тиреотоксикоза, где одновременно под наблюдением офтальмолога будет проводиться диагностика и лечение ЭОП. Командная работа очень важна, т.к. эффективность лечения ЭОП будет зависеть от быстроты достижения стойкого эутиреоидного состояния, точности определения активности и тяжести ЭОП, наличия осложнений, требующих оперативного лечения.

В развитии ЭОП выделяют две основные фазы. В первой фазе активного воспаления происходит нарастание симптомов ЭОП, затем следует фаза плато, когда симптомы активности сохраняются, но не прогрессируют, затем симптомы регрессируют, и процесс становится неактивным, при этом могут сохраняться зрительные нарушения и косметические дефекты. Определение активности ЭОП очень важно с клинической точки зрения, т.к. от активности процесса зависит выбор лечения и тактика ведения пациента.

Описан клинический случай этапного лечения ЭОП, осложненной оптической нейропатией и глазодвигательными нарушениями, резистентной к иммуносупрессивной терапии глюкокортикоидами и потребовавшей глубокой латеральной декомпрессии орбит, у пациентки с болезнью Грейвса.

## Abstract (en)

According to modern concepts, thyroid eye disease (TED) is an independent progressive autoimmune disease of the organ of vision, closely associated with the autoimmune pathology of the thyroid gland (TG), (ICD code — H06.2, proptosis in case of impaired thyroid function E05.0). TED treatment is a long step-by-step process, including immunosuppressive therapy, radiation therapy of the orbits and surgical treatment.

TED is a multidisciplinary problem. A patient with thyrotoxicosis clinic and TED symptoms will be taken to an endocrinological clinic for normalization of thyroid hormones and treatment of thyrotoxicosis complications. At the same time, under the supervision of an ophthalmologist, TED diagnostics and treatment will be carried out. Teamwork is of utmost importance because the effectiveness of TED treatment will depend on the speed of achieving a stable euthyroid state, the accuracy of determining the TED activity and severity, and the presence of complications requiring surgical treatment.

There are two main phases in the TED development. In the first phase of active inflammation, an increase in the symptoms of TED occurs, then a plateau phase follows, when the symptoms of activity persist but do not progress, then the symptoms regress and the process becomes inactive, while visual disturbances and cosmetic defects may persist. Determining the TED activity is very important from a clinical point of view, because the choice of treatment and tactics of patient management depend on the inflammation activity.

We describe a clinical case of phasing treatment of TED complicated by optic neuropathy and movement disorders in a patient with Graves’ disease, resistant to immunosuppressive therapy with glucocorticoids and requiring deep lateral bony orbital decompression.

## АКТУАЛЬНОСТЬ

ЭОП является наиболее частой и тяжелой аутоиммунной патологией, осложняющей течение болезни Грейвса (БГ). В большинстве случаев ЭОП протекает без серьезных зрительных расстройств, однако примерно у 40–45% становится тяжелой и требует хирургического лечения с целью коррекции зрения и косметических дефектов, у 5% пациентов развивается оптическая нейропатия (ОН), требующая проведения срочной костной декомпрессии. Это может быть связано с несвоевременной диагностикой как БГ, так и ЭОП, неправильным определением фазы ЭОП, несвоевременным и ненадлежащим лечением.

Лечение ЭОП у пациентов с БГ представляет собой длительный многоэтапный процесс. Если ЭОП манифестировала одновременно с БГ, первым этапом является нормализация уровня тиреоидных гормонов: свободных фракций тироксина и трийодтиронина (свТ4 и свТ3). Если ЭОП развилась после манифестации БГ, проводится коррекция антитиреоидной терапии. Через 2–3 недели начинается второй этап — лечение ЭОП. При наличии ЭОП, осложненной ОН с угрозой потери зрения, лечение БГ и ЭОП проводится одновременно. Лечение ЭОП начинается с проведения иммуносупрессивной терапии глюкокортикоидами (ГК). При неэффективности иммуносупрессивной терапии ГК рассматривается проведение лучевой терапии орбит на фоне введения ГК в уменьшенной дозе или без ГК. При сохранении ОН выполняется следующий этап — костная декомпрессия орбит и, при необходимости, последующая хирургическая реабилитация [1]. Мы представляем клинический случай пациентки с тяжелой ЭОП и сложным выбором персонализированной терапии.

## КЛИНИЧЕСКИЙ СЛУЧАЙ

Пациентка Т, считает себя больной с ноября 2019 г., когда стала обращать внимание на отечность век. Через месяц стали беспокоить сердцебиение, раздражительность, дрожь в теле, снижение веса. В январе 2020 г. диагностирована БГ, инициирована терапия тирозолом в дозе 10 мг. В августе 2020 г. терапия тирозолом была отменена в связи со стабильным течением заболевания. Однако в связи с ухудшением состояния через неделю терапия тирозолом возобновлена.

Ухудшение со стороны глаз отметила в октябре 2020 г., консультирована офтальмологом по месту жительства, проведены ретробульбарные инъекции дексаметазона, но без особого эффекта. Доза тирозола менялась от 5 мг до 10 мг. Проведено МРТ орбит от 11.12.2020 г.: экзофтальм, утолщение всех прямых и верхней косой мышцы, признаки отека экстраокулярных мышц (ЭОМ) и ретробульбарной клетчатки (РБК), апикальное сгущение.

Консультирована офтальмологом ФГБУ «НМИЦ эндокринологии» 29.12.2020 г. Заключение: ЭОП тяжелой степени, активная фаза (по шкале клинической активности CAS=6/7), осложненная двусторонней ОН, частичной офтальмоплегией. Ретракция век, хемоз, лагофтальм, кератопатия. Острота зрения (далее везде — максимально корригированная острота зрения): правый глаз=0,6, левый глаз =0,8. По рекомендации офтальмолога амбулаторно проведены инъекции метилпреднизолона в режиме пульс-терапии в суммарной дозе 5750 мг.

В феврале 2021 г. госпитализирована в ФГБУ «НМИЦ эндокринологии» (рис. 1). На фоне приема тирозола — эутиреоидное состояние (ТТГ — 1,48 мкМЕ/мл). При проведении УЗИ выявлены два образования в левой доле ЩЖ: на границе с перешейком — изоэхогенное образование размером 1,8х1,7х1,3 см и в н/полюсе — частично загрудинное изоэхогенное образование неоднородной структуры с анэхогенными включениями, кальцинатом по центру размером 2,9х2,4х1,6 см (EU-TIRADS 3). По заключению цитологического исследования: фолликулярная неоплазия (Bethesda IV) и коллоидный узел (Bethesda II). Рекомендовано оперативное лечение в объеме тиреоидэктомии в плановом порядке [2].

**Figure:**
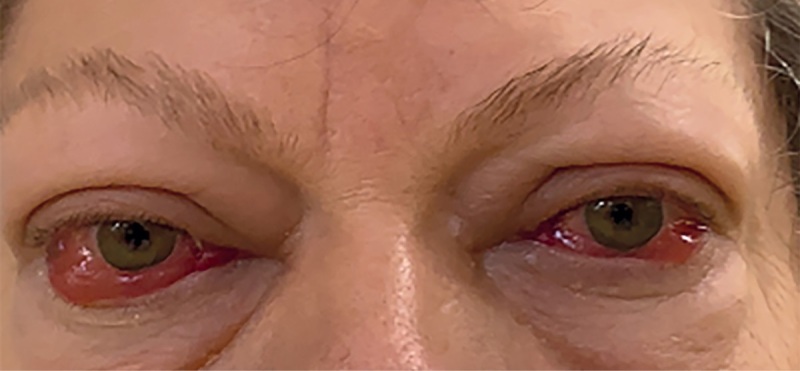
Рисунок 1. Внешний вид пациентки при поступлении в “НМИЦ эндокринологии”. ЭОП тяжелой степени, активная фаза, осложненная ОН, отеком век, выраженным красным хемозом, лагофтальмом, кератопатией.

По данным МРТ орбит: без существенной динамики по сравнению с МРТ от 11.12.2020 г. (рис. 2 А, В, С). По заключению офтальмолога: ЭОП тяжелой степени, активная фаза (CAS=4); двусторонняя ОН; частичная офтальмоплегия; ретракция век; хемоз; лагофтальм. Острота зрения: правый глаз=0,4, левый глаз=0,6. С учетом сохраняющейся активности ЭОП и прогрессирующего снижения зрительных функций продолжена пульс-терапия ГК (суммарная доза метилпреднизолона 5750 мг + преднизолона 1800 мг). На этом фоне достигнут положительный эффект — улучшение подвижности глаз и уменьшение отека век. Однако, по данным компьютерной периметрии (Humphrey 30-2), сохранялись зрительные расстройства: правый глаз — диффузное снижение контрастной светочувствительности, множественные абсолютные скотомы в нижней половине, относительные скотомы в верхней половине, левый глаз — диффузное снижение контрастной светочувствительности, множественные относительные скотомы, в связи с чем пациентка была направлена на консультацию в ФГБНУ НИИ ГБ им. Краснова для решения вопроса о хирургическом лечении ЭОП (декомпрессии орбит).

**Figure:**
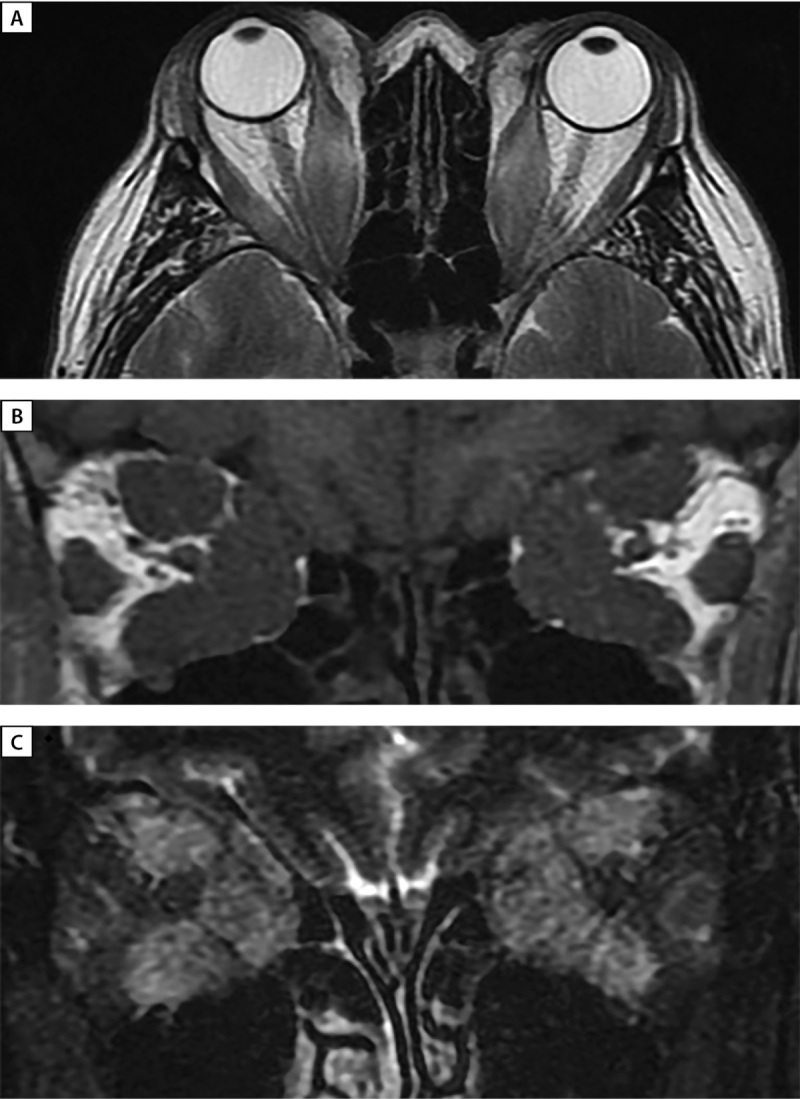
Рисунок 2. МРТ орбит (А — аксиальная; В, С — корональная проекции) до лечения: выраженное утолщение верхних, нижних, медиальных прямых мышц, апикальный синдром. С — режим Т2 STIR c жироподавлением — выраженный отек всех ЭОМ.

В связи с наличием ОН и миогенной формы ЭОП выполнены глубокие костные декомпрессии латеральной стенки обеих орбит (КДО) (правой орбиты — 10.03.2021 г., левой орбиты — 16.03.2021 г.). Особенностями оперативного лечения было применение ультразвукового остеодеструктора Sonopet Ultrasonic Aspirator (Stryker Medtex, Япония), которым проводилось дополнительное расширение размера первичного костного окна в среднем на 5 мм с формированием скошенного профиля его заднего края (патент на изобретение RU2742799) и обнажением твердой мозговой оболочки средней черепной ямки (рис. 3). В послеоперационном периоде отмечали повышение остроты зрения на правом глазу с 0,4 до 0,8; на левом — с 0,6 до 0,8. Также наблюдали положительную динамику по показателям компьютерной периметрии (Octopus 600) в виде повышения светочувствительности, сокращения числа абсолютных и относительных скотом: средний порог светочувствительности (MS) повысился с 17,1 до 25,7 dB; дефект светочувствительности (MD) снизился с 8,1 до -0,7 dB; глубина скотом (sLV) уменьшилась с 5,7 до 4,4 dB (рис. 4).

**Figure:**
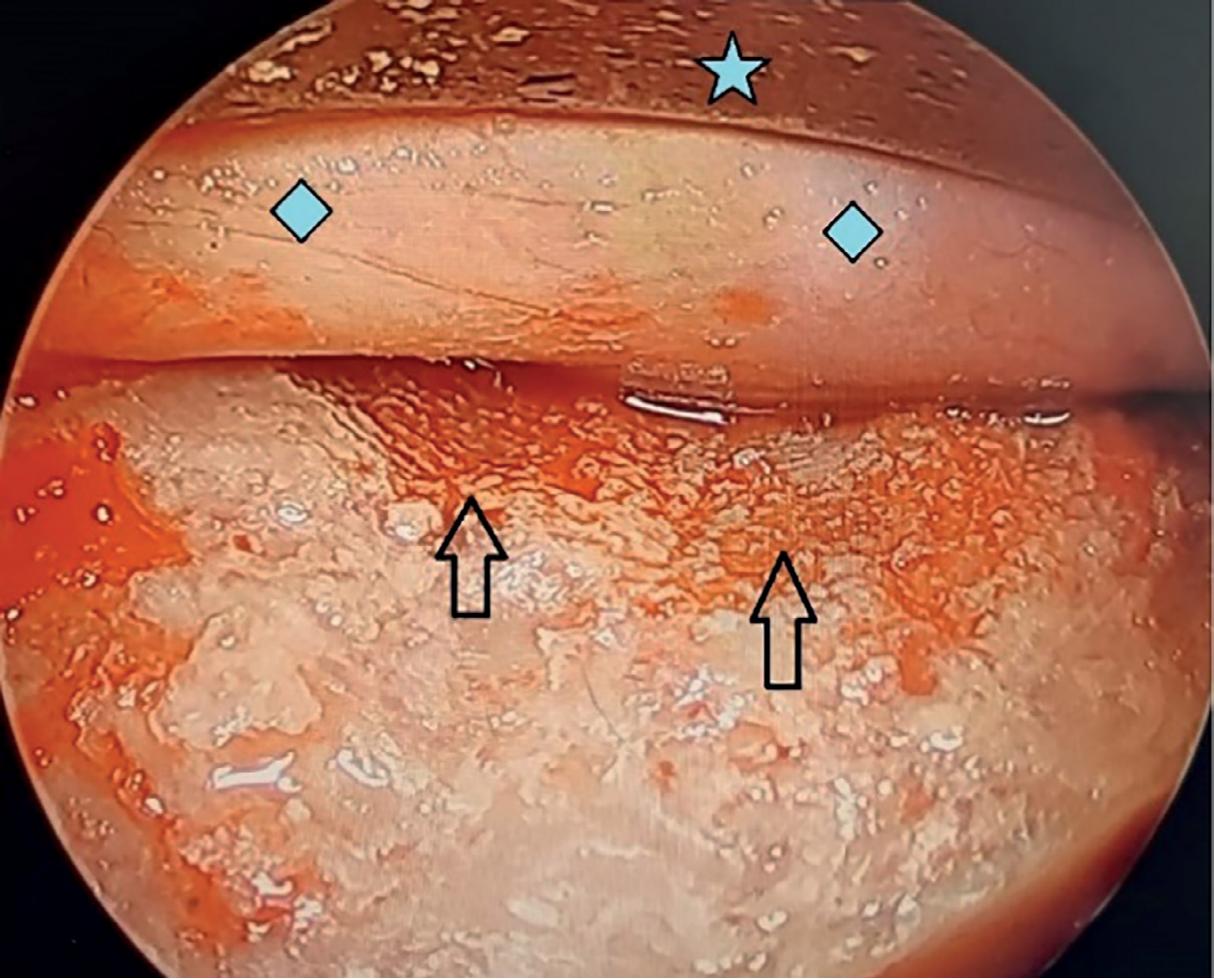
Рисунок 3. Интраоперационное фото. По краям зоны обнажения ТМО визуализируются следы ультразвуковой остеодеструкции (стрелки). Надкостница орбиты (). Орбитальное зеркало (звездочка).

**Figure:**
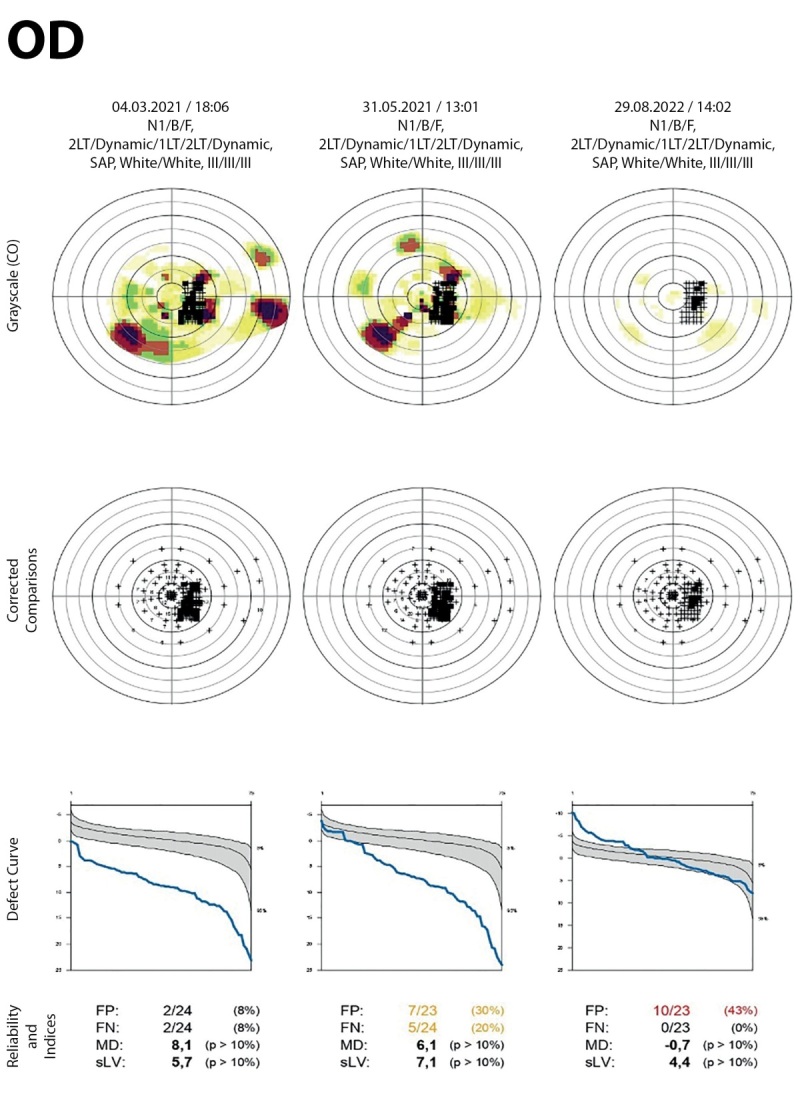
Рисунок 4. Динамика показателей компьютерной периметрии (Octopus 600) правого глаза до, через 2 месяца и 17 месяцев после латеральной КДО: средний порог светочувствительности (MS) повысился с 17,1 до 25,7 dB; дефект светочувствительности (MD) снизился с 8,1 до -0,7 dB; глубина скотом (sLV) уменьшилась с 5,7 до 4,4 dB.

В июле 2021 г. пациентка повторно госпитализирована в ФГБУ «НМИЦ эндокринологии». По заключению офтальмолога: ЭОП тяжелой степени, активная фаза (CAS=4). ОН; частичная офтальмоплегия; ретракция век; лагофтальм (рис. 5). Состояние после пульс-терапии ГК в суммарной дозе метилпреднизолона 7750 мг + преднизолона 3600 мг (декабрь 2020 г. — март, июль 2021 г.), глубокой декомпрессии латеральной стенки обеих орбит. Острота зрения: правый глаз=0,7, левый глаз=1,0. В связи с продолжающейся активностью ЭОП (рис. 6 А, В) рекомендовано продолжить проведение пульс-терапии преднизолоном (1500 мг введено во время госпитализации) далее продолжено в амбулаторном режиме в дозе 600 мг 1 раз в неделю 4 капельницы (2400 мг).

**Figure:**
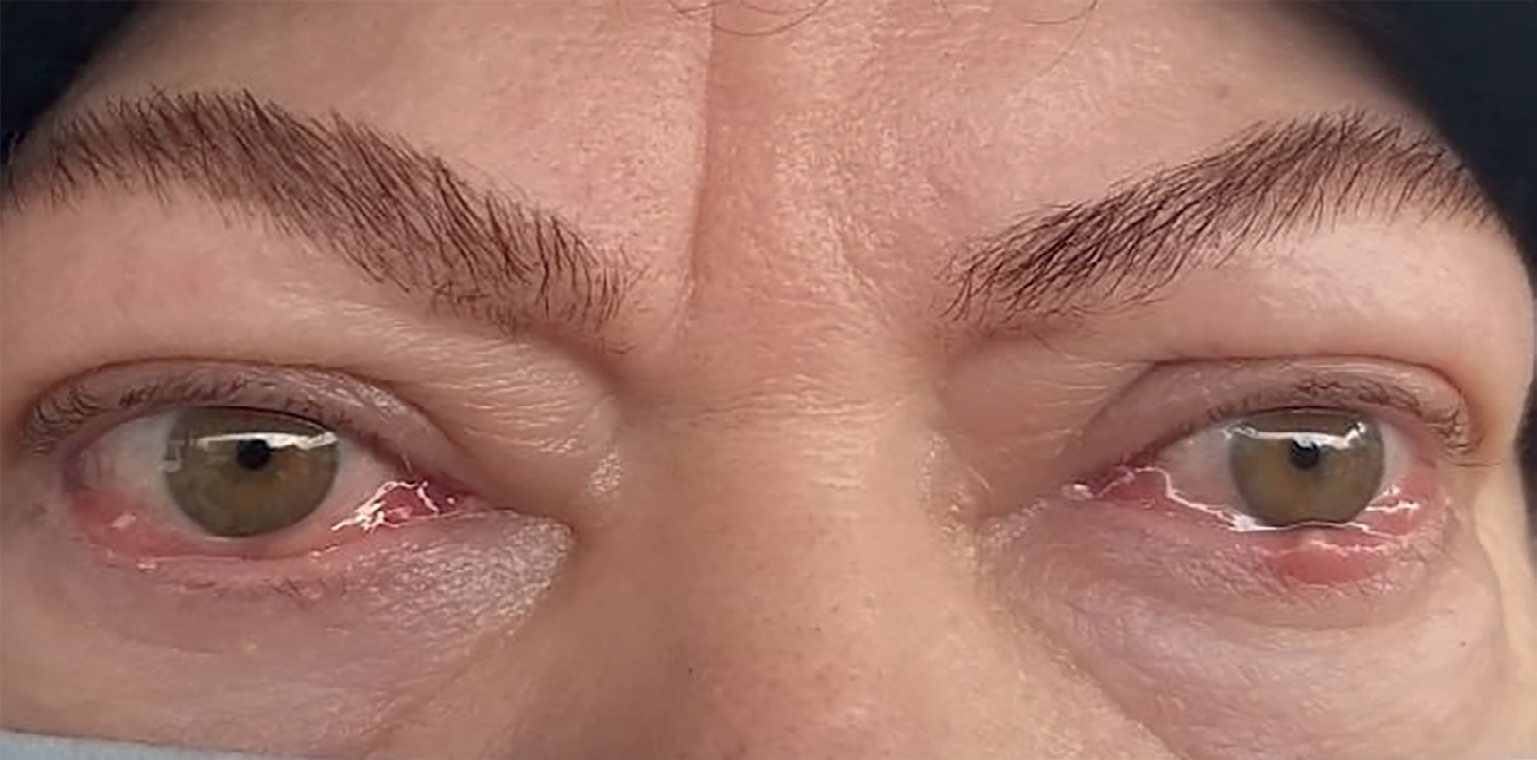
Рисунок 5. Внешний вид пациентки через 4 месяца после глубокой латеральной КДО с двух сторон. Экзофтальм уменьшился на 4 мм, значимая положительная динамика по большинству симптомов, включая хемоз и другую воспалительную симптоматику.

**Figure:**
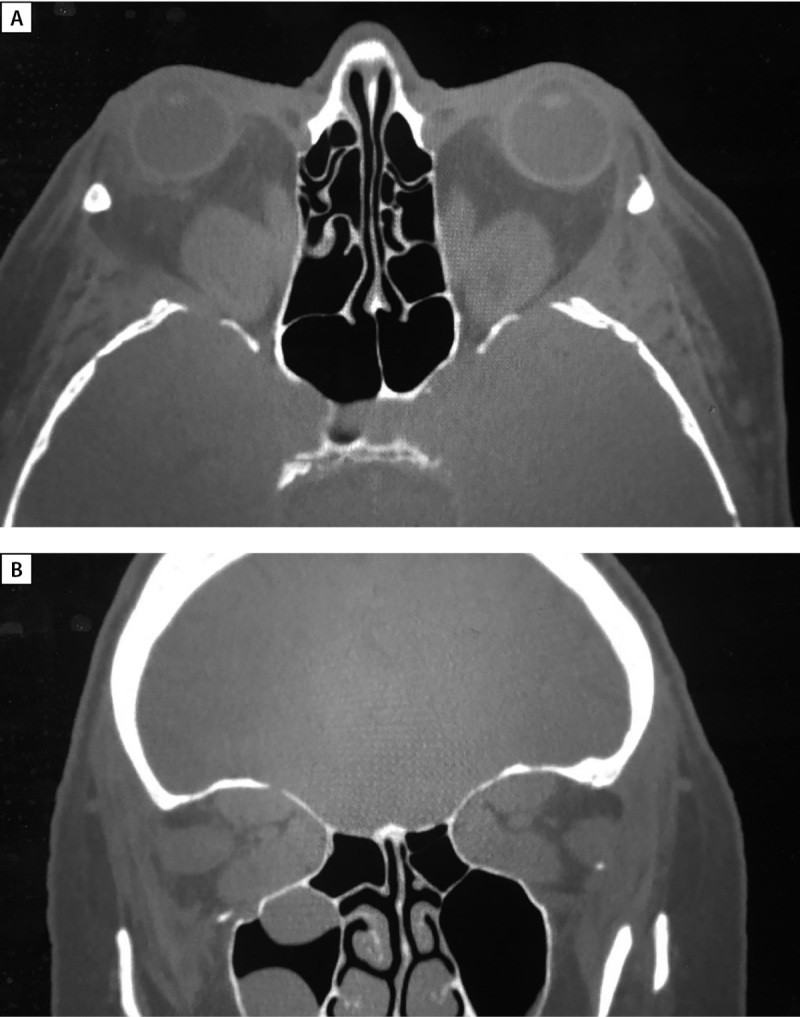
Рисунок 6. МСКТ орбит (А — аксиальная, В — корональная проекции) через 4 месяца после оригинальной методики глубокой латеральной КДО с двух сторон. Определяются послеоперационные дефекты латеральных стенок орбиты, вплоть до обнажения ТМО средних черепных ямок, смещение наружных прямых мышц и орбитального жира в область дефектов соответствующих стенок.

Учитывая возраст, введение мегадоз ГК, пациентка консультирована на предмет возможных побочных эффектов от системной глюкокортикоидной терапии [3]. Уточнено наличие сопутствующих заболеваний: гипертоническая болезнь II, стадия АГ 2, риск ССО 3. AV блокада 1 ст. Гастроэзофагеальная рефлюксная болезнь. Поверхностный антральный гастрит. Неалкогольная жировая болезнь печени: жировой гепатоз. Ожирение II ст. Нарушенная гликемия натощак. Дорсопатия. Полиостеоартроз. Мышечно-фасциальный синдром на фоне вертебропатии. Миома матки. Пролапс тазовых органов: опущение передней и задней стенок влагалища I ст. На фоне проводимой антигипертензивной терапии показатели АД, глюкозы, печеночных проб в пределах околоцелевых.

В ФГБУ «НМИЦ эндокринологии» 14.12.21 г. выполнена экстрафасциальная тиреоидэктомия. По данным морфологического исследования послеоперационного материала: папиллярная карцинома левой доли ЩЖ; pT1bNxMx; узловой коллоидный зоб. В послеоперационном периоде инициирована гормональная терапия левотироксином натрия в дозе 100 мкг. После выписки доза левотироксина увеличена до 150 мкг. Паратгормон от 20.01.2022 — 2,5 пмоль/л (1,6–6,9), фосфор — 1,33 ммоль/л (0,74–1,52), 25(ОН) D — 39 нг/мл, кальций ионизированный — 1,14 ммоль/л.

В апреле 2022 г. при обследовании в ФГБУ «НМИЦ эндокринологии» подтверждена компенсация первичного гипотиреоза (ТТГ — 3,38 мМЕ/л) на фоне приема левотироксина натрия в дозе 150 мкг/сут. Данных за рецидив папиллярной карциномы не получено (тиреоглобулин 0,065 нг/мл, антитела к тиреоглобулину — 28,98 МЕ/мл). По данным МРТ орбит: двусторонний экзофтальм, утолщение, жировая трансформация ЭОМ. Умеренный отек нижних прямых мышц и правой верхней прямой мышцы. Уплотнение, незначительный отек РБК с обеих сторон. Пациентка осмотрена офтальмологом: острота зрения обоих глаз=1.0, CAS=3, рекомендован динамический контроль.

В августе 2022 г. при очередном обследовании в ФГБУ «НМИЦ эндокринологии» на фоне приема левотироксина натрия в дозе 150 мкг/сут сохранялся эутиреоз. Данных за рецидив папиллярного рака не получено (тиреоглобулин — 0,014 нг/мл, антитела к тиреоглобулину — 18,49 МЕ/мл, норма 0–115) (рис. 7). По данным МРТ орбит: утолщение, жировая трансформация ЭОМ, умеренный отек нижних прямых и правой верхней прямой мышцы. Уплотнение, незначительный отек РБК с обеих сторон (рис. 8 А, В). По заключению офтальмолога: ЭОП неактивная фаза (CAS=1). Острота зрения обоих глаз=1,0. Состояние после пульс-терапии ГК в суммарной дозе: метилпреднизолон 7750 мг + преднизолон 5100 мг (декабрь 2020 г. — март, июль–август 2021 г.). Пациентка продолжает наблюдаться в ФГБУ «НМИЦ эндокринологии» и в ФГБНУ НИИ ГБ им. М.М. Краснова.

**Figure:**
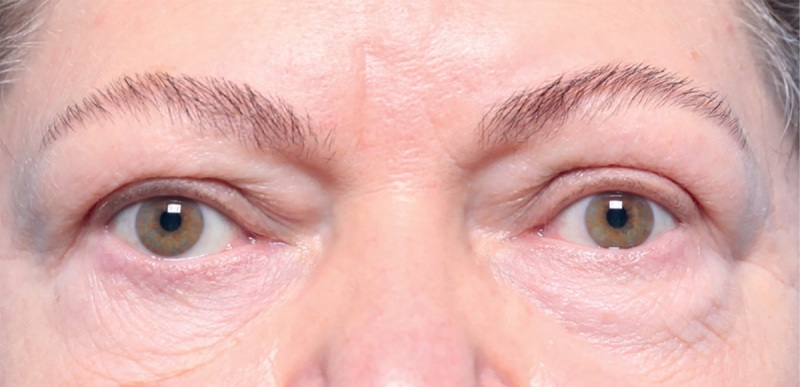
Рисунок 7. Через 16 месяцев после глубокой латеральной КДО с двух сторон признаки активности ЭОП отсутствуют.

**Figure:**
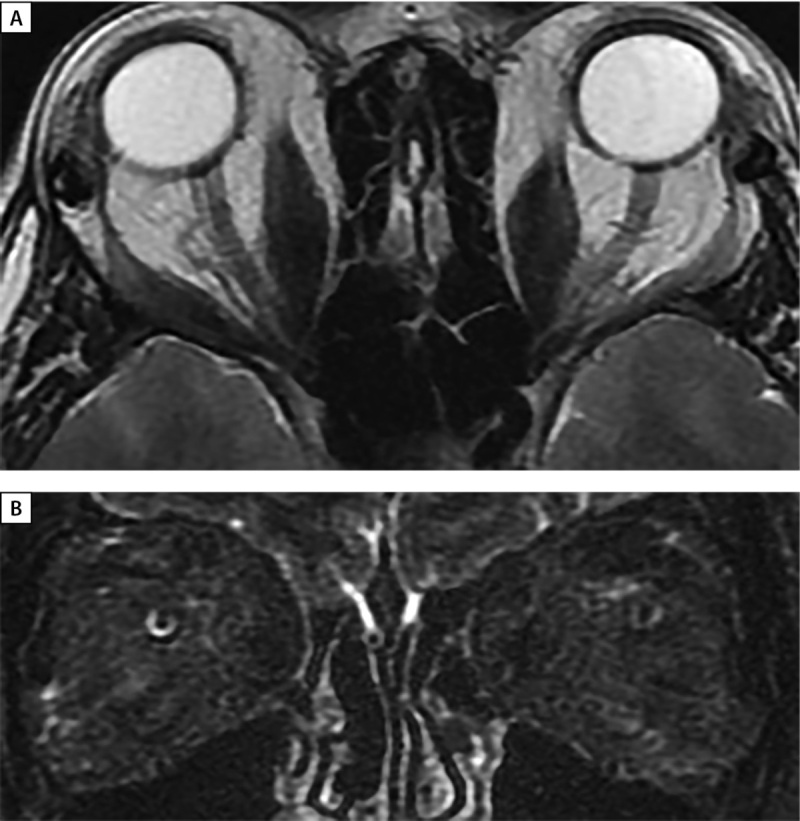
Рисунок 8. МРТ орбит через 16 месяцев после глубокой латеральной КДО с двух сторон. А — аксиальная проекция — выход мягких тканей орбиты в область латеральной остеотомии, отмечается уменьшение в объеме ЭОМ. В — корональная проекция, режим Т2 STIR c жироподавлением отсутствует отек ЭОМ и РБК.

## ОБСУЖДЕНИЕ

ЭОП является наиболее частой и тяжелой аутоиммунной патологией, осложняющей течение БГ. Лечение ЭОП включает много этапов, а также участие разных специалистов: эндокринологов, офтальмологов, офтальмохирургов, эндокринных хирургов, кардиологов. Лечение ЭОП, развившейся на фоне манифестации БГ или в процессе ее лечения, начинается у эндокринолога с компенсации тиреотоксикоза или коррекции проводимой терапии. Одновременно проводится оценка тяжести и активности ЭОП и необходимости проведения срочных мероприятий, направленных на сохранение зрения. Наиболее опасным, угрожающим зрению осложнением ЭОП является ОН, которая развивается примерно у 5% пациентов, и при ненадлежащей диагностике и отсутствии своевременного лечения может привести к стойкому снижению зрительных функций и слепоте. Лечение активной ЭОП всегда начинается с иммуносупрессивной терапии ГК в режиме пульс-терапии или при невозможности ее выполнить с назначения пероральных ГК. При резистентности к ГК и угрозе прогрессирования ОН вторым этапом является проведение костной декомпрессии орбит [4]. Орбитальная декомпрессия — это хирургический метод лечения, направленный на увеличение орбитального объема и/или уменьшение объема орбитального жира. Для пациентов с рефрактерностью к лечению ГК декомпрессия костной стенки является ключевым этапом лечения. Улучшение зрительных функций отмечается у 82% пациентов на следующее утро после декомпрессии орбиты [5–7].

Особенностью представленного клинического случая было проведение операции на латеральной, а не медиальной стенке орбиты, что обычно является методикой выбора при ОН. Выбор стенки был обусловлен топографическими особенностями орбит, в частности длиной стенки, очень высоким риском получить большую девиацию после декомпрессии медиальной стенки [7–10]. Глубокая латеральная КДО была выполнена в близком к максимальному объему с обнажением твердой мозговой оболочки. Этот принципиальный этап был выполнен специальным ультразвуковым наконечником, позволяющим выполнять остеодеструкцию в опасной зоне особо деликатно, исключая возможность травматизации мягких тканей орбиты [11][12]. Таким образом, глубокая КДО, проведенная по оригинальной методике, позволила добиться значительной положительной динамики по зрительным функциям, а последующая парентеральная терапия сопровождалась восстановлением зрения до 1.0 и восстановлением периферического зрения (средний порог светочувствительности (MS)=25,7dB; дефект светочувствительности (MD)=-0,7 dB; глубина скотом (sLV)=4,4 dB).

## ЗАКЛЮЧЕНИЕ

Данный клинический случай представляет интерес в связи с тяжелым течением рефрактерной ЭОП, которая привела к развитию ОН. На примере этого случая можно продемонстрировать многоэтапность и преемственность в лечении ЭОП. Поскольку ЭОП была активной, первой линией лечения стала пульс-терапия ГК. Однако ввиду ее недостаточной эффективности и прогрессирования ОН была выполнена глубокая латеральная костная декомпрессия орбиты с каждой стороны для увеличения объема костной орбиты, улучшения кровообращения и разгрузки структур орбиты, в первую очередь зрительного нерва. Лечение ЭОП почти непрерывно продолжалось в течение двух лет. Второй особенностью явилась выявленная в процессе обследования папиллярная карцинома ЩЖ, которая была оперирована уже после проведения иммуносупрессивной терапии, костной декомпрессии и снижения активности ЭОП. Итогом последовательно проводимого персонализированного лечения стало восстановление зрения до 1.0 с коррекцией и устранение косметических дефектов, а также отсутствие прогрессирования онкопроцесса в ЩЖ.

## Дополнительная информация

Источник финансирования. Работа выполнена при поддержке Министерства образования и науки (грант РНФ №22-15-00135. Научное обоснование, разработка и внедрение новых технологий диагностики коморбидных йододефицитных и аутоиммунных заболеваний щитовидной железы, в том числе с использованием возможностей искусственного интеллекта).

Конфликт интересов. Авторы декларируют отсутствие явных и потенциальных конфликтов интересов, связанных с публикацией настоящей статьи.

Участие авторов. Все авторы одобрили финальную версию статьи перед публикацией, выразили согласие нести ответственность за все аспекты работы, подразумевающую надлежащее изучение и решение вопросов, связанных с точностью или добросовестностью любой части работы.
